# ßNp63 controls cellular redox status

**DOI:** 10.18632/oncoscience.217

**Published:** 2015-08-25

**Authors:** Margherita Annicchiarico-Petruzzelli, Nicola Di Daniele, Eleonora Candi

**Affiliations:** University of Rome “Tor Vergata”, Department of Experimental Medicine and Surgery, Rome, Italy

**Keywords:** oxidative stress, p63

A widely accepted hypothesis indicates that the accumulation of oxidative damage arising from endogenously produced reactive oxygen species (ROS) is one of the main triggers of cellular senescence, age-related disorders and cancer development. High ROS levels are responsible for lipid peroxidation, protein, and DNA oxidation, thus seriously threatening cellular fate and enhancing mutations risks [1]. The regulation of redox homeostasis is crucial to maintain a normal cellular function, ensuring cell proliferation and survival. An important protein, which is able to sustain epithelial proliferation/survival to counteract cellular senescence and organismal aging, is the transcription factor p63 [2]. TP63 belongs to the p53 gene family; it is expressed as multiple isoforms for the presence of two N-terminus promoters and C-terminus alternative splicing [2]. The ßNp63 isoforms lack the N-terminal transactivation domain but they are still able to activate transcription of selected genes, using a second transactivation domain. ßNp63 is responsible for activation of different cellular programs controlling cell proliferation potential and stemness, epithelial stratification, cell–cell, and cell-matrix adhesion [2, 3]. Interestingly, depletion of ßNp63 in primary human keratinocytes and in cancer cell lines (lung, breast) increases endogenous ROS levels [4] suggesting that p63-dependent mechanisms are required in epithelial cells to control redox status. A recent study identified cytoglobin (CYGB) as a novel ßNp63 target gene [4]. The role of CYGB in cellular metabolism is partially unknown, its expression increases under oxygen deficiency and oxidative stress. It binds oxygen, therefore it may facilitate oxygen diffusion to mitochondria. It detects O_2_ concentration in cells and protects them from ROS [4]. CYBG is co-expressed with ßNp63 in the basal layer of the epidermis, the proliferative compartment, and *in vitro*, in proliferating keratinocytes. Down-modulation of CYGB in keratinocytes increases endogenous ROS levels, with consequent p53-mediated induction of apoptosis. The CYGB protecting role is also more evident upon oxidative stress, as CYGB-silenced keratinocytes undergoing to oxidative stress results in elevated level of ROS accumulation that parallel high level of apoptotic events. These results indicate that in physiological conditions, ßNp63, via CYGB, plays an important role in maintaining physiological redox status. Thus, this could be one of the mechanisms engaged by ßNp63 [4, 5], to counteract senescence/ageing and maintain high proliferative potential in epithelial stem cells.

The redox status of cancer cells differs from that of normal cells and the modulation of oxidative stress is very relevant in both tumor formation and response to anti-cancer therapy. Indeed, high ROS levels in cancer cells are consequence of alteration in several signalling pathways that affect cellular metabolism. These ROS levels are balanced to elevated anti-oxidant defence pathways in cancer cells. Therefore, the role of ROS in tumorigenesis is still under debate: the increased anti-oxidant capacity suggests that high ROS level could contribute to a barrier against tumorigenesis, on the other hand, ROS may promote tumorigenesis by inducing DNA mutations and pro-oncogenic signalling [6–8]. The ßNp63-CYGB axis could be part of this scenario, controlling cellular redox state in cancer cells. Indeed, experimental evidences show that CYGB-silenced lung SCC cells exhibit elevated ROS levels leading to apoptosis as well as an increased sensitivity to doxorubicin treatments [4]. Furthermore, computational analysis of lung cancer data sets, indicates that ßNp63 and CYGB co-expression is a negative prognostic marker for patients, showing significant survival reduction. Glutathione peroxidase (GPX2) is another ßNp63 direct target, which inhibits the activation of p53 by reducing the extent of oxidative stresses and oxidative stress-induced apoptosis in cancer cells [4].

In summary, ßNp63 maintains redox cellular status in physiological condition, by direct transcriptional control of CYGB and GPX2, possibly preventing senescence/ageing and maintaining epithelial stem cells. Given that ßNp63 is frequently amplified in tumors and acts in a dominant-negative manner over p53, ßNp63 action as a pro-survival factor is, at least in part, mediated by the inhibition of the p53-dependent oxidative stress-induced apoptotic response. Furthermore, the ßNp63-antioxidant properties may modulate therapeutic efficiency of anticancer treatments that act directly and/or indirectly regulating ROS levels.
